# SARS-CoV-2 air and surface contamination in residential settings

**DOI:** 10.1038/s41598-022-22679-y

**Published:** 2022-10-27

**Authors:** Gil Correia, Luís Rodrigues, Mariana Afonso, Marta Mota, Joana Oliveira, Rui Soares, Ana Luísa Tomás, Anna Reichel, Patrícia M. Silva, José J. Costa, Manuel Gameiro da Silva, Nuno C. Santos, Teresa Gonçalves

**Affiliations:** 1 FMUC, Faculty of Medicine, Univ Coimbra, Rua Larga, 3004-504 Coimbra, Portugal; 2 Medical Microbiology Research Group, CNC-Center for Neurosciences and Cell Biology, 3004-504 Coimbra, Portugal; 3 ARS Centro, IP, Alameda Júlio Henriques, 3000-457 Coimbra, Portugal; 4 Universitary Clinic of Nephrology, Faculty of Medicine University of Coimbra Nephrology Service, Hospital and University Center of Coimbra, Coimbra, Portugal; 5 Department of Clinical Pathology, Instituto Português de Oncologia de Coimbra Francisco Gentil EPE, 3000-075 Coimbra, Portugal; 6 Instituto de Medicina Molecular, Faculdade de Medicina, Universidade de Lisboa, Av. Prof. Egas Moniz, 1649-028 Lisbon, Portugal; 7 ADAI, Department of Mechanical Engineering, Univ Coimbra, Rua Luís Reis Santos, Pólo II, 3030-788 Coimbra, Portugal

**Keywords:** Viral infection, Air microbiology

## Abstract

SARS-CoV-2 transmission occurs mainly indoors, through virus-laden airborne particles. Although the presence and infectivity of SARS-CoV-2 in aerosol are now acknowledged, the underlying circumstances for its occurrence are still under investigation. The contamination of domiciliary environments during the isolation of SARS-CoV-2-infected patients in their respective rooms in individual houses and in a nursing home was investigated by collecting surface and air samples in these environments. Surface contamination was detected in different contexts, both on high and low-touch surfaces. To determine the presence of virus particles in the air, two sampling methodologies were used: air and deposition sampling. Positive deposition samples were found in sampling locations above the patient’s height, and SARS-CoV-2 RNA was detected in impactation air samples within a size fraction below 2.5 μm. Surface samples rendered the highest positivity rate and persistence for a longer period. The presence of aerosolized SARS-CoV-2 RNA occurred mainly in deposition samples and closer to symptom onset. To evaluate the infectivity of selected positive samples, SARS-CoV-2 viability assays were performed, but our study was not able to validate the virus viability. The presented results confirm the presence of aerosolized SARS-CoV-2 RNA in indoor compartments occupied by COVID-19 patients with mild symptoms, in the absence of aerosol-generating clinical procedures.

## Introduction

Human-to-human SARS-CoV-2 transmissions have been reported since, at least, mid of December 2019^1,2^ and COVID-19 was declared a pandemic on March 11, 2020, by the World Health Organization (WHO). The debate over the modes of transmission and their relative importance was extensive and is still ongoing, with broad implications on control measures^3,4^. A growing body of evidence has emerged concerning the airborne transmission potential for SARS-CoV-2 aerosols^5–12^. In early 2020 the WHO assumed that “close contact” with infected individuals is a critical risk factor for acquiring the infection and recognized transmission through aerosols in crowded indoor spaces, although not excluding transmission by droplets or fomites^13^. Since May 2021, the Centers for Disease Control and Prevention (CDC) has also assumed the possibility of transmission through aerosol particles containing SARS-CoV-2^14^. Measures to mitigate the spread of COVID-19, such as global lockdowns and patient isolation/quarantine, were the most reliable contribution to limiting the virus’s spread in the community. Vaccines were introduced in December 2020, further minimizing the transmission chains of SARS-CoV-2. All these measures highlighted the role of household transmission, indicating that spread occurs mainly in indoor settings that favor close contact between people^15,16^.

Respiratory viruses spread through four main routes: direct physical contact, indirect contact from fomites, large droplets, and fine aerosols^17^. Air transmission encompasses distinct modes of acquiring the infection, depending on particle size. Traditionally, droplet transmission refers to the possibility that particles larger than 5 µm containing the virus may serve as vehicles for interpersonal spread. Interestingly, however, there may not be such a clear size threshold although it is accepted that larger particles tend to fall near the emitter, at a distance that depends on the balance between the aerodynamic forces, dependent on the particle size, shape and initial velocity, and the gravitational force^18^. On the other hand, particles smaller than 5 µm have a different behavior, staying airborne and traveling longer distances. These small-size virus-laden particles represent true aerosol transmission particles^10^. Such particles can be liquid or solid and, when suspended, may remain airborne for seconds to hours, being capable of traveling hundreds of meters. However, a clear distinction between droplet and aerosol transmission is difficult to unveil^3,4,6,10,19–21^.

The formation of virus-containing aerosols may occur directly from infected patients or by mechanical means, when air currents disperse the virus into the air from contaminated surfaces^21^. Aerosols can be produced and dispersed by individuals in everyday activities, such as breathing, speaking, or singing^10,13,22^.

Under laboratory conditions, the virus remains detectable and viable for several days on different surfaces^23–25^. The viability decay directly correlates with increasing temperature and is also influenced by other factors such as humidity level, exposure to UV rays, type of material and surface roughness^10,12,23–26^. Viable virus has been isolated in the air surrounding infected patients and viral RNA was identified in aerosol particles more than 1.5 m away from their source^12,19,27^. Poor ventilation in confined indoor spaces is associated with increased transmission of respiratory infections and detection of virus in mechanical ventilation systems further demonstrates airborne transmission, highlighting the need for an adequate implementation of ventilation systems to contribute to protecting against airborne infections^20,28,29^.

Extensive environmental contamination with the virus in facilities hosting infected patients, both in the air and on surfaces, is well documented and poses substantial risks for susceptible individuals^12,20,27,30–35^. There is mounting evidence on the contamination of the spaces surrounding self-isolated and mildly ill patients^36–46^. In Portugal, in the years 2020 and 2021, home isolation and care were advised for individuals with a high-risk contact, suspected or confirmed infection with asymptomatic or mild to moderate disease^36^. In such a setting, it is expected that households would continue to account for a significant proportion of infections^37^. Other residential settings, such as care homes, have been one of the most prominent sources of outbreaks, with devastating consequences^15,38–41^. Similarly to hospital facilities, environmental contamination with SARS-CoV-2 in the spaces housing the infected individuals is expected^42^. Therefore, it is crucial to assess viral dissemination routes in residential spaces without mechanical ventilation, to better understand the mechanisms and patterns of viral shedding and contagion.

This work is focused on the characterization of SARS-CoV-2 shedding and persistence in the environment surrounding asymptomatic or mildly ill SARS-CoV-2-infected patients under home or residential care. Four selected COVID-19 confirmed or suspected patients under domiciliary care were enrolled in a Portuguese Primary Care Unit in the center of Portugal for household contamination assessment, between November 2020 and January 2021. A section of infected patient bedrooms in a nursing home with an outbreak of SARS-CoV-2 infection in February 2021 was also analyzed. Surface and air samples were collected in both settings and analyzed for detection of SARS-CoV-2 RNA and viral viability.

## Results

### Sampling sites: households

The 4 analysed households lived in very different conditions. Figure 3 shows an overview of the sampling areas and sites of the 4 households (A, B, C, and D). Positive samples were obtained in houses A and B. Samples from house D were cultured for SARS-CoV-2 detection, but did not demonstrated viral presence. The complete gathered datasets regarding sampling characterization are shown in the Supplementary Material.

In House A, 4 out of 5 surface samples were positive, and collected from a laptop, TV remote control, toilet flusher button, and bathroom door handle. All air samples were negative. However, SARS-CoV-2 RNA was detected in deposition samples obtained on the 9^th^ day after symptom onset, both in the living room and in the bedroom.

In House B SARS-CoV-2 RNA was detected in the air samples collected on the same day of the patient’s positive test, using Impactor, for particles below 2.5 μm, over an extended sampling period of 22 h. Air samples collected on the subsequent days did not show the presence of viral RNA. Viral RNA was detected on the surfaces of the patient’s laptop, kitchen door handle, bathroom light switch, and the flusher button. Additionally, one deposition sample collected in the living room was positive for SARS-CoV-2.

In house C, sampling occurred on two separate occasions, 7 and 8 days after diagnosis. All environmental samples were negative in house C.

All environmental samples were negative in house D. Air samples were cultured within 6 h after the collection period. However, SARS-CoV-2 RNA presence was likewise not detected.

In houses A, C, and D, infected patients were confined to one room. Transmission events within households occurred only in house A, where transmission most likely occurred already before the domiciliary quarantine. Positive surface and deposition samples were detected in houses A and B. In house B, SARS-CoV-2 RNA was present in a segregated air sample in the aerosol size range (< 2.5 µm).

### Sampling sites: nursing home

**Figure 1:**
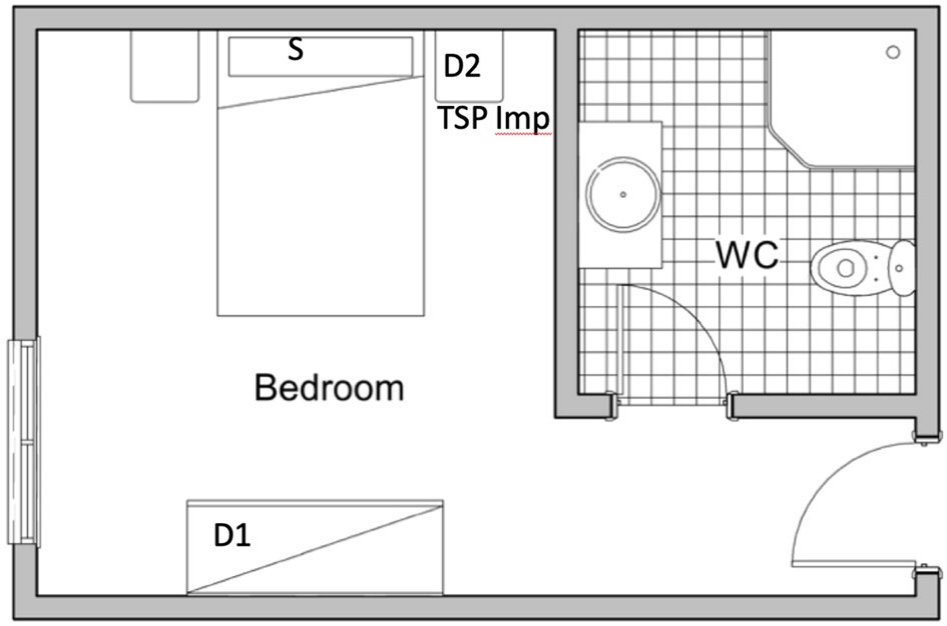
Schematic top view illustration of the rooms plants and representative sampling sites, similar over the 4 nursing rooms (AA, BB, CC and DD). *D1, D2* Deposition samples sites, *S* surface samples, *TSP* total suspended particles, *Imp.* impactor air samples.

In the nursing home, all bedrooms had similar configurations, Fig. 1 shows a scheme with the sampling locations inside the bedroom where positive air samples were detected. Deposition samples collected from all 12 bedrooms on the same date were negative for the presence of viral RNA over an exposure period of 18.5 h.

Room AA was occupied by two SARS-CoV-2-infected female patients of 92 and 84 years old. Sampling was conducted on day 3 of the symptom onset of the 84-year-old patient. In Room AA viral RNA was detected in a single swab sample from the TV remote control. All other surface, deposition, and air samples were negative (in a total of 9 samples). Room BB was occupied by a 93-year-old man. The two surface samples collected were positive for SARS-CoV-2. One was taken from a highly touched surface—the top of a refrigerator, located near the bed (height ~ 0.8 m)—and the other from an untouched surface, the top of a bedside bookcase (height ~ 1.8 m). Deposition and air samples were negative in this room.

In room CC, sampling occurred 1 day after the diagnostic PCR test of a positive 92-year-old bedridden patient that was relocated on the same day to this room, therefore, surface samples were not collected. Although the patient was symptomatic, both deposition and air samples turned out negative.

The patient from room DD was an 89-year-old woman. Deposition samples were negative and a single swab sample from the bed rail was positive. The detection of viral RNA in the air occurred in a 20-min sampling period during usual hygiene care and diaper change on the 3^rd^ day after diagnosis. The sampler was placed on the bedside table. A total of 10 air samples were collected; however, no other positive samples were detected. Of those, culture was attempted, however, no viral replication was observed.

### SARS-CoV-2 detection

All environmental samples were studied by RT-PCR to detect viral RNA. Results are given for Gene E, because of increased sensitivity. Further analysis results are available in Supplementary Material (Supplementary Tables S1, S2). Table 1 summarizes the results of sampling across all spaces.

**Table 1:** Results from house sampling (houses A, B, C and D) and from nursing home sampling (rooms AA, BB, CC, DD).

House/room	Air		Deposition		Surface	
	Location/type	Gene E	Location/Height (m)	Gene E	Location	Gene E
A	Bedroom + WC (TSP)	Neg	Bedroom (1.8)	**33.89**	Laptop^a^	**23.12**
	Bedroom (> 2.5))	Neg	Living room (1.8)	**34.66**	Toilet^a^ flusher	**33.38**
	Bedroom (2.5–1)	Neg			WC door handle^b^	**32.29**
	Bedroom (1–0.50)	Neg			TV^b^ remote	**35.01**
	Bedroom (0.50–0.25)	Neg			Laptop^c^	Neg
	Bedroom (> 2.5)	Neg				
	Bedroom (< 2.5)	Neg				
	Living room (TSP)	Neg				
B	Living room (> 2.5)	Neg	Bedroom (2)	**35.28**	Laptop	**27.19**
	Living room (< 2.5)	**34.81**	Living room (2)	neg	WC switch	**30.48**
	WC (TSP)	Neg			Kitchen door handle^a^	**28.22**
	Living room (TSP)	Neg			Toilet flusher^a^	**32.76**
	Living room (> 2.5)	Neg				
	Living room (< 2.5)	Neg				
C	Corridor (TSP)	Neg	WC (1.6)	Neg	WC door handle	Neg
	Living room (> 2.5)	Neg	Bedroom (1.8)	Neg	Laptop	Neg
	Living room (< 2.5)	Neg	Living room (1.6)	Neg	Bedside table	Neg
	Bedroom (TSP)	Neg	Corridor (1.2)	Neg	Mobile phone	Neg
D	Bedroom (> 2.5)^d^	Neg	Bedroom (0.8)	Neg	Bedside table	Neg
	Bedroom (< 2.5)^d^	Neg	Corridor (0.8)	Neg	Bedroom door handle	Neg
	Bedroom (TSP)	Neg			Bedroom switch	Neg
					TV remote	Neg
AA	Bedroom (TSP)	Neg	Bedroom (1)	Neg	TV remote	**36.98**
	Bedroom (> 2.5)	Neg	Bedroom (1)	Neg	Bedside table	Neg
	Bedroom (< 2.5)	Neg	Bedroom (1)	Neg	Bed footboard	Neg
BB	Bedroom 1.8 (TSP)	Neg	Bedroom (1.8)	Neg	Bedside fridge (0,8)	**30.87**
			Bedroom (1)	Neg	Bed footboard (0.8)	**35.98**
CC	Bedroom (TSP)	Neg	Bedside table (0.8)	Neg		
	Bedroom (TSP)	Neg				
DD	Bedroom (TSP)	Neg	Bedroom (1)	Neg	Bedside table	Neg
	Bedroom (> 2.5)^d^	Neg	Bedroom (1)	Neg	Bed footboard (0.8)	**34.85**
	Bedroom (< 2.5)	Neg	Bedside table (0.8)	Neg		
	Bedroom (TSP)	Neg	Bedside table (0.8)	Neg		
	Bedroom (TSP)^d^	Neg	Bedroom (1)	Neg		
	Bedroom (TSP)	**36.19**				
	Bedroom (TSP)^d^	Neg				
	Bedroom (TSP)	Neg				
	Bedroom (TSP)^d^	Neg				
	Bedroom (TSP)	Neg				

*Gene E* cycle threshold for RT-PCR, *Neg* negative result, undetected, *TSP* total suspended particles, > *2.5* impactor sample from stage A, < *2.5* impactor sample from stage B + C + D.

Significant values are given in bold.

^a^Cotton.

^b^Gauze.

^c^PTFE filter.

^d^Air samples used for viability assays.

**Figure 2:**
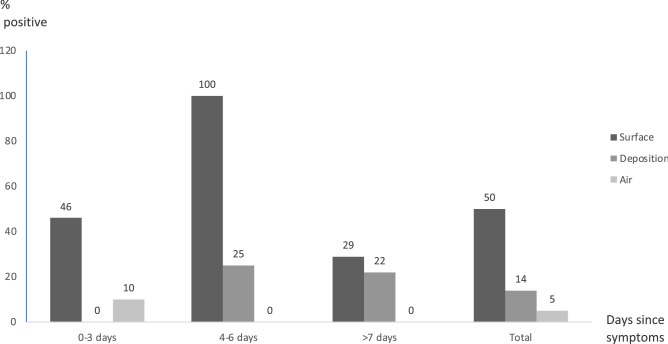
Distribution of total positive samples from the spaces studied according to the number of days after onset of symptoms.

Table 2 lists the gender, age, and main symptoms (temperature and cough) of the patients, together with the collection date and days from symptoms onset, as well as the positive results from the surface, air, and deposition samples. Results are provided as the number of positive samples per total number for each sample type. Figure 2 summarizes the overall positivity rate, by period of sampling, for all samples from houses and selected rooms of the nursing home.

**Table 2:** Summary of samples, patients’ characterization and collection method.

Patients^a^		Collection date		Days since symptoms	Fever^b^	Cough	Presence of SARS-CoV-2			Total
							Surface	Deposition	Air^c^	
A	M46	05/Nov		3	No	No	**4**/5	–	0/5	6/15
		09/Nov		7	No	No	–	–	0/2	
		11/Nov		9	No	No	–	**2**/2	0/1	
B	F25	10/Nov		1	Yes	No	–	–	**1**/2	6/12
		12/Nov		4	No	No	**4**/4	–	0/2	
		13/Nov		5	No	No	–	1/2	–	
		16/Nov		8	No	No	–	–	0/2	
C	F22	16/Dec		7	No	No	0/4	0/4	0/3	0/12
		17/Dec		8	No	No	–	–	0/1	
D	F36	20/Jan		1	Yes	No	0/4	0/2	0/3^c^	0/9
AA	F84	16/Feb		3	No	Yes	**1**/2	–	0/2	1/9
		17/Feb		4	No	Yes	–	–	0/1	
		18/Feb		5	No	Yes	–	0/2	–	
		26/Feb		13	No	No	0/1	0/1	–	
BB	M93	16/Feb		13	No	Yes	**2**/2	0/1	0/1	2/5
		18/Feb		15	No	Yes	–	0/1	–	
CC	M92	16/Feb		1	Yes	Yes	–	–	0/1	0/3
		17/Feb		2	Yes	Yes	–	–	0/1	
		18/Feb		3	Yes	Yes	–	0/1	–	
DD	F89	18/Feb		no	No	No	–	0/2	–	2/17
		26/Feb		1	Yes	No	**1**/2	0/3	0/4	
		27/Feb		2	Yes	No	–	–	0/1	
		28/Feb		3	Yes	No	–	–	**1**/2^c^	
		01/Mar		4	Yes	No	–	–	0/3^c^	
Total				Mean	Fever	Cough	Surface	Deposition	Air	Total
				5.3	37.5%	33.3%	12/24	3/21	2/37	17/82

Significant values are given in bold.

^a^Gender (male—M or female—F) and age of each patient are indicated.

^b^Patient body temperature above 38 °C.

^c^Air samples used for viability assays.

During this work, a total of 82 samples were collected, during different periods after symptom onset, for the enrolled study patients. Thereby, the highest number of positive air samples was taken in the first period (0–3 days) after symptom onset (10%), while in the following period (4–6 days) all surface samples tested positive. The deposition samples rendered positive results within the different time periods considered (Fig. 2).

All positive air samples were tested for viral viability. However, no SARS-CoV-2 replication in host cells was confirmed after RT-PCR of the most promising supernatants.

## Discussion

Global preventive measures against SARS-CoV-2/COVID-19 led to a shift of transmission from public to domestic settings. In a systematic review and meta-analysis, the transmission rate depended on the symptom status of the index case, and was higher for symptomatic than for asymptomatic households^37^. Multiple evidences pointed towards the presence of SARS-CoV-2 RNA on household surfaces and in wastewater, but not in air samples^43,44^. The determination of air contamination is essential to address prevention measures to protect households of quarantined individuals and caregivers. The sampling strategy of the present study aimed to detect SARS-CoV-2 in domestic, non-mechanically ventilated environments, housing mildly symptomatic patients. Our study demonstrated the presence of SARS-CoV-2 viral RNA in air, deposition, and surface samples from 4 houses with COVID-19 patients in domiciliary care and 4 different rooms inside a nursing home housing infected patients.

We analyzed the spaces housing patients that met the inclusion criteria in 4 different households. Patients’ age ranged from 22 to 46 years old, and all presented mild symptoms. The collection period ranged from the 1^st^ to the 9th day of symptoms. Among the studied patients, two had no significant comorbidities (patients B and C), while patient A had obesity, hypertension, dyslipidemia and hyperuricemia, and patient D had obesity and hypercoagulability. Symptoms did not include cough and the 2 patients diagnosed with fever had a maximum temperature ranging from 37.4 to 38.6 °C, with fever lasting for a single day.

Sampling in the nursing home included patients with secondary cases after the first cluster of detected infections. As all patients were isolated in individual or double rooms, detected viral RNA would correspond to the infected patient. Samples were obtained in different periods for each patient and preferably within the first days after diagnosis. Viral shedding from infected individuals is more likely to occur within this period and correlates with the viral load detected by RT-qPCR in patients’ nasal swabs^45–47^. Nevertheless, sampling occurred in some occasions several days after symptom onset that makes viral detection more unlikely, especially in the case of air samples. We encountered heterogeneous contamination with SARS-CoV-2 RNA among the housing spaces of infected patients, according to the different sampling types. The highest detection rate was obtained for surface samples, followed by deposition samples, and finally air samples.

Surface contamination is expected to be due to direct and indirect deposition of the virus^20,48^. Samples from personal objects such as TV remote controls or other touched surfaces like bed rails were positive and suggest direct contact, while positive samples from elevated untouched surfaces suggest deposition and persistence of detectable virus. Surface samples had a higher positivity rate, compared to the other types of sampling, as previously described^24,33,49^. Curiously, the positivity rate of surface samples is lower after the 7th day from symptom onset (Fig. 2). Houses C and D were the only spaces where surface samples were obtained and none was positive. However, the heterogeneity found in these samples may be due to different disinfection routines, which were not assessed, and not only due to distinct patterns of viral shedding.

Despite the greater positivity rate in the surface sampling, our assessment of infectivity was limited, as culture was not attempted in surface samples. Since the viability of the virus on surfaces is already well documented^25^, we focused on studying viral viability on the collected positive aerosol samples.

In this study, we found SARS-CoV-2 RNA in deposition samples placed at several locations and heights inside the spaces accommodating the patients. Positive deposition samples were retrieved from Petri dishes placed above patients’ height, which makes droplet contamination less likely. Typically, droplets describe a ballistic-type trajectory after emission and deposit nearby and below the source of emission^22,50^. Viral detection in these samples reinforces that suspended particles may be an important route for surface contamination, in addition to direct contact by the patients. Therefore, it is likely that the contamination observed can represent a cumulative contamination of the environment and, thus, would be lower on the first days of symptoms. Taken together, our results support this hypothesis. The detected RNA may correspond to different sizes of virus-laden particles and variable rates of emission and decay.

Despite the initial results of positive deposition samples in houses A and B, no further viral RNA was detected in subsequent sampling. This is consistent with higher contamination verified in both environments. We would expect positive samples at least in the DD room where the virus was retrieved from the air, however, factors such as opening windows that could decrease the viral load in the air or disinfection were not controlled. Furthermore, the positive air sample was collected for a short period, during hygiene, and may correspond to aerosolization from the deposited virus. In the rooms where positive surface samples have been found and none of the deposition samples were positive, we hypothesize that positive samples are the result of previous shedding.

Air sampling poses many difficulties. Depending on particle´s size, the mechanism of capture needs to be different^51^. SARS-CoV-2 RNA was detected by RT-PCR both with long and short sampling periods. However, the detection rate was low. This can be explained by low concentration in the sampled air^27,30^, low capture efficiency, low sampling air volume (= volume flow rate × sampling time) and/or losses during sampling and sample processing, namely by the adhesion of particles to the interior walls of the cassettes mediated by electrostatic forces. Estimations of viral aerosolization demonstrate that variable degrees of shedding are expected to occur^52^. The air sampler location in the room was variable and so was the distance to the patient, as the placement was intended not to interfere with regular activities and autonomous patients moved freely. With the sampling methods used in this study, we were able to detect suspended viral RNA in two occasions, one in a segregated air sample and the other in the total suspended particles. Nevertheless, we believe the height at which the samplers were placed (from 1.8 to 2 m) and the fact that some samples tested positive are results of utmost importance. The latter results are consistent with the shedding of virus-containing aerosol as the dispersion of such particles, from an individual, in a calm atmosphere, is influenced by a body-to-air temperature gradient, known as human thermal plume, that contributes to the ascension of exhaled respiratory particles^53^.

The heterogeneous environmental contamination observed is consistent with other sampling studies and with the heterogeneity of infectivity observed among family members and households^27,35,42,54^. Nevertheless, this can result from several different factors besides quantitative differences in viral shedding. A single set of samples in the cases of negative results could not have detected viral shedding in a different period. Furthermore, shedding characteristics will depend on the viral load and region where viral replication is more active: droplets are more likely to be produced in the upper airways by strong airflows, such as upon coughing, sneezing or talking, while airborne sized particles formation is more expected to occur in the lower airways^10^.

Overall, air samples were positive on two occasions in the same 24 h period and in a short 20 min total suspended particles (TSP) sample during hygiene. The presence of SARS-CoV-2 RNA under such conditions may be the result of either direct exhaled aerosols or aerosolization from deposited aerosols on bed linen or from diaper content. The latter would be consistent with extensive contamination observed in toilets^44,55,56^. The detection of the virus in a relatively small period of sampling suggests larger quantities of suspended particles containing the virus or specific conditions affecting air dissemination^4^.

The detection of SARS-CoV-2 RNA by RT-PCR does not prove infectivity. Although multiple aspects of the pandemic suggest airborne transmission, there is limited evidence for viable virus particles detected in air^12,57,58^. To address this issue, we attempted culture in selected air samples. However, supernatant analysis by RT-PCR was unable to detect viral RNA. Notwithstanding, multiple factors can contribute to this result, which does not prove the virus is unable to remain viable in aerosols. Loss of viability can be due to the extensive sampling period, as it can lead to desiccation of the viral particle. A low density of virus in the air is expected and the virus recovery from the filter can further contribute to its inactivation^21^.

Our work demonstrates multiple environmental contaminations from SARS-CoV-2 infected patients in different types of domiciliary spaces housing infected patients. All patients had a mild form of COVID-19, were not receiving oxygen therapy and no aerosol-generating procedure was performed. Nevertheless, we have documented evidence for the presence of SARS-CoV-2 RNA in aerosols in the different sampled environments. Viral detection occurred for all sample types: surfaces, deposition and air. This finding highlights the possibility of aerosolization and transmission of SARS-CoV-2 without any aerosol-generating procedures. Viral RNA presence in the different contexts and, especially, the presence of viral RNA in air samples during hygiene care, raise particular concerns for susceptible households and caregivers.

Viral shedding has been observed in the first days of infection and, when detected, the presence of SARS-CoV-2 is often consistent across different types of samples. Our findings demonstrate that shedding occurs through both direct and indirect contact, from the deposition of air-suspended particles containing the virus. The detection of viral RNA in the air from a height above of the emission suggests that the virus can remain airborne and travel long distances from its emission source. This would not be possible in the case of exclusive virus-containing droplet-sized particles. However, only small amounts of virus were detected in air samples. Moreover, the presence of viral RNA is insufficient to prove infectivity, and our assays with air samples were unable to demonstrate the presence of viable virus. Still, our findings confirm the presence of the virus and elicit the need to address direct actions toward preventing airborne infection outside hospital settings.

## Materials and methods

We conducted an observational study and undertook all procedures without interfering with the patients' daily living activities. Sampling occurred from November 2020 to February 2021 on multiple separate occasions. Different spaces housing COVID-19 patients in individual houses and bedrooms of a nursing home were analyzed for viral contamination. The material and equipment preparation, as well as the subsequent sanitization, were performed in the laboratory. All non-disposable material employed for sampling or sample processing was disinfected with isopropyl alcohol by immersion for, at least 24 h, and deionized water was used to remove excess alcohol afterwards.

### Patients’ selection and enrollment

Adult patients with SARS-CoV-2 infection, confirmed by RT-PCR, and validated by a reference laboratory in accordance with the Portuguese health authority regulating the diagnosis of SARS-CoV-2 infection (Direção Geral de Saúde, DGS), were asked to volunteer for this study, and enrolled upon signing an informed consent. Cycle threshold (Ct) for nasopharyngeal swab samples PCR are given whenever molecular analysis was carried in our laboratory. Sampling occurred preferably early after diagnosis or symptoms onset, however, in two cases sampling was performed 13 and 15 days after symptom onset. On the first sampling day, surface, air, and deposition samples were obtained from each environment. Whenever viral contamination was detected, further samples were collected in the subsequent days. Autonomous patients moved freely during the sampling period and no guidance was made concerning the use of masks. After collection, samples were sent to the lab within 2 h and immediately processed and/or stored at -80 °C.

The complete gathered datasets regarding patients’ characterization are shown in Table 3.

**Table 3:** Patients demographics and characterization.

Patient	A	B	C	D	AA	BB	CC	DD
*CT*^a^	21.1	20.27	33.67^b^	21.91^b^				
Age	46	27	22	35	84	93	92	89
Gender^c^	M	F	F	F	F	M	M	F
Weight (kg)	95	72	58	128	66	77	55	40
Height (m)	1.77	1.74	1.71	1.66	1.66	1.85	1.68	1.55
Body Mass Index (kg/m^2^)	30.3	23.8	19.8	46.5	24.0	22.5	19.5	16.6
Symptom onset	Nov 02, 2020	Nov 09, 2020	Dec 09, 2020	Jan 19, 2021	Feb 13, 2021	Feb 03, 2021	Unknown	Unknown
Diagnosis	Nov 02, 2020	Nov 10, 2020	Dec 12, 2020	Jan 20, 2021	Feb 15, 2021	Feb 05, 2021	Feb 15, 2021	Feb 25, 2021
Max. Temp. (°C)	38.0	37.4	no	38.6	38.2	38.6	38.8	38.0
Cough	no	no	no	yes	yes	yes	yes	no
Corticoids	no	no	no	no	no	no	no	no
Comorbidities	Hypertension; dyslipidaemia; hyperuricemia; gallbladder lytiasis	0	0	Obesity. Coagulopathy (elevated Factor VIII, VWF)	Hypertension; Type 2 Diabetes; Glaucoma	Hypertension; dyslipidaemia;	Hypertension; Stroke;	Hypertension; dyslipidaemia; Cardiac valvular disease; Alzheimer;
Medication/vaccine	Simvastatin 20 mg; Alopurinol, 300 mg; Perindopril + Indapamide, 4 mg + 1.25 mg + ursodeoxycholic acid	0	0	aspirin/dipyridamole 25/200 mg	vaccine (1st dose 21/01/2021)	vaccine (1st dose 21/01/2021)	vaccine (1st dose 21/01/2021)	vaccine (1st dose 21/01/2021)

^a^CT for Gene E, exceptions are marked.

^b^CT for Gene N.

^c^Male—M or female—F.

### Location

In this work, two different environments accommodating quarantined COVID-19 patients were sampled for SARS-CoV-2 air and surface contamination. A total of 8 infected subjects were included, 4 of them from different isolated households, and the other from 4 individual patient rooms in a nursing home with a SARS-CoV-2 outbreak. Patients’ demographics and characteristics are presented in Table 3. For both environments, surface, deposition, and air samples were collected and analyzed. Moreover, the configuration of the households and the nursing home rooms were taken into consideration for the interpretation of the results.

Houses A and B corresponded to apartments, while C and D were 2-floor single family homes.

**Figure 3:**
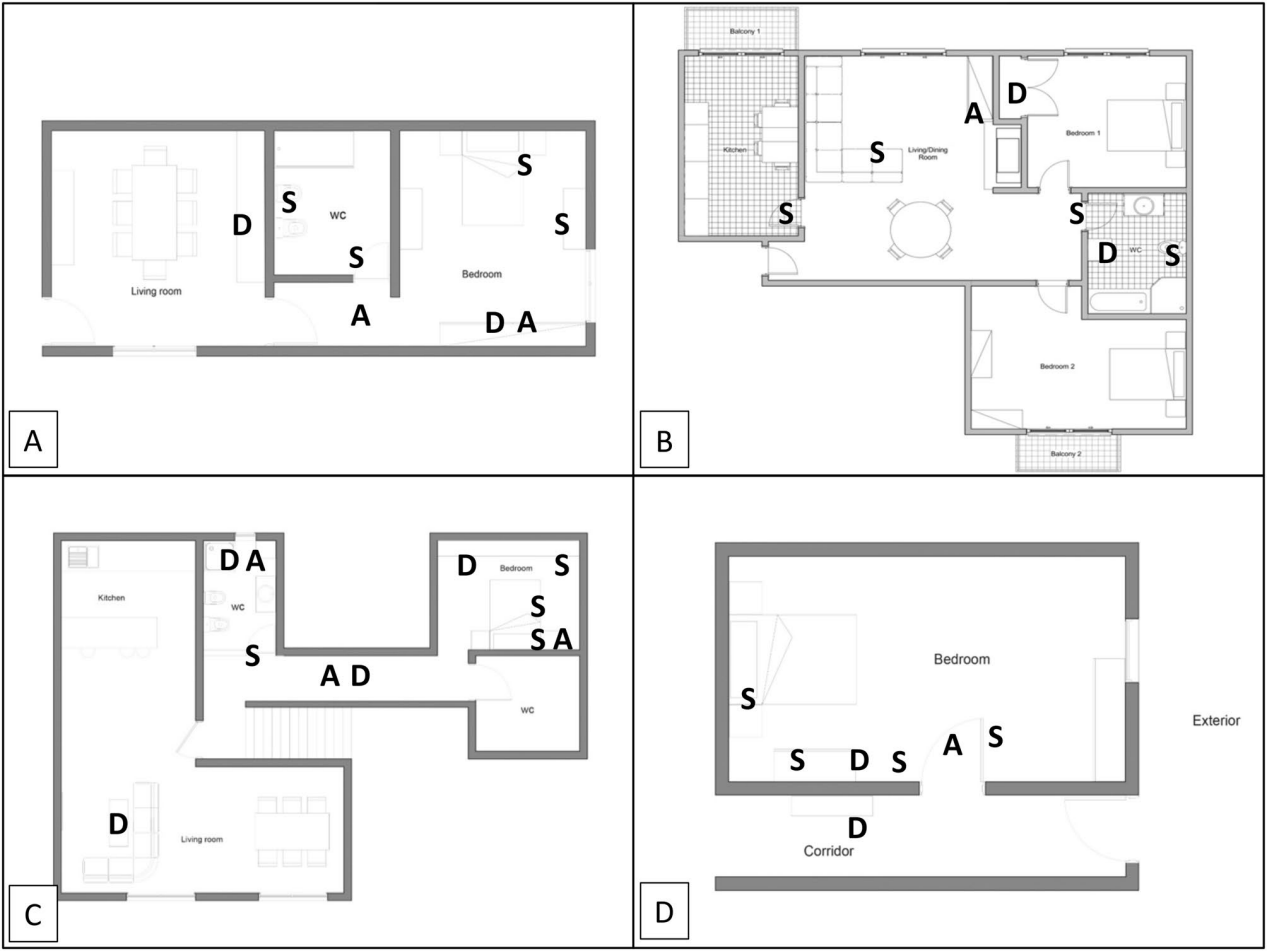
Schematic top view illustration of the representative house areas and sampling sites. The spaces where sampling occurred are designated accordingly (Houses A, B, C and D). *A* air samples, *S* surface samples, *D* deposition samples.

In house A, the patient was isolated in a bedroom with a private bathroom, fully separated from his spouse and their 2 children, living in a two-bedroom apartment. The first set of samples included air and surfaces and was collected from the bedroom on the 3rd day after the patient´s symptom onset. The patient’s spouse started to develop symptoms on the 4th day after his original onset. Surface samples were collected from a laptop, TV remote control, toilet flusher button, and bathroom door handle. Air and deposition samples were collected in the bedroom and in the living room, on top of the furniture, at a height above 1.6 m.

House B was likewise a two-bedroom apartment, occupied by a single person. The patient was prescribed an antigen test that confirmed the diagnosis on the day after symptom onset. Surface samples included personal items and surfaces. Air samples were collected from the living room and bathroom and deposition samples, placed at a height above 1.8 m, were retrieved from the bedroom and the living room after a 67-h period.

In house C, the patient lived with her mother, who was not infected, during the duration of this study. The patient was isolated in one room with a shared bathroom. Air sampling cassettes were placed in the corridor between the bedroom and the bathroom, and a segregated air sample was taken inside the bathroom. Deposition samples included different locations inside the house, while surface sampling included only personal or close objects, as most of the surfaces and objects were thoroughly and frequently disinfected.

The patient in house D lived with her husband and 2 daughters, none of whom got infected, during the duration of this study.”. She was isolated within a bedroom, but had to share the bathroom. Collection of samples occurred on the first day after fever onset. Except for one deposition sample collected from the corridor outside the bedroom, all other were taken from inside the bedroom.

A total of 18 residents inhabited the nursing home at the moment of the first confirmed case of COVID-19. Patients were moved from their original rooms and divided into two separate wings: a contaminated area for positive patients and another for residents who tested negative. As a sampling strategy, 4 rooms with infected patients were selected for air and surface sampling and named AA, BB, CC and DD (Table 3). In addition, deposition samples were collected from all nursing home bedrooms on the same date (18^th^ of February).

### Surface sampling

The studied surfaces included personal objects, such as TV remote control or personal computers, and highly touched and untouched surfaces, such as bed rails or above-height top of furniture. Sterile nylon flocked swabs were used for sampling and placed immediately, after collection, in sterile screw-cap transport tubes with 2 mL of Inactivation Transport Medium (ITM), containing Hanks balanced salt solution, fetal bovine serum, antibacterial antibiotics, antifungal antibiotics and phenol red (Wuxi NEST Biotechnology, China). In House A, 4 cm^2^ of gauze or cotton and one Whatman 47 mm, 0.2 μm PTFE filter were employed in surface sampling. Swab surface sample areas were variable, depending on the surface sampled.

### Air sampling

For direct virus detection in the air, different types of air samples were collected. For air capture, two methods were used: filtration and impaction. TSP and size-segregated aerosol samples were collected. In both cases, gelatin filters with a pore size of 3 μm (Sartorius, Germany), which are suitable for the recovery of much smaller-sized particles, such as viruses, including SARS-CoV-2 or bacteriophages^27,59^, were employed. TSPs were collected on a 25- or 37-mm-diameter gelatin filter that was placed in a clear styrene filter cassette (SKC Inc., UK). Size segregated aerosol samples were collected using a miniature cascade impactor (Sioutas impactor, SKC Inc., UK) loaded with 25 mm gelatin filters for all 4 impactation stages. The latter allows particle separation by their aerodynamic size into four size ranges (0.25–0.50 μm, 0.50–1.0 μm, 1.0–2.5 μm, and > 2.5 μm). To increase concentration and probability of viral detection, collection filters for particles below 2.5 μm were processed together (exceptions are indicated). Stage A (> 2.5 μm) and B–C–D (< 2.5 μm) samples were treated separately.

A Leland Legacy Personal Sample Pump (SKC Inc., UK) was used at a flow rate of 5 L/min for TSP samples and 9 L/min for size-segregated samples. An AirChek XR5000 Pump (SKC Inc., UK) was used for TSP samples at 5 L/min. Pumps were calibrated before sampling with a Drycal airflow meter (Defender 510, Mesa Labs, USA) to adjust the flow rates to nominal flow within ± 5% range. For processing, gelatin filters were dissolved in ITM or Viral Transport Medium (VTM) when samples were used for infectious assays. VTM was prepared according to CDC specifications, using Hanks balanced salt solution with calcium and magnesium ions, no phenol red, heat-inactivated fetal bovine serum, gentamicin sulfate, and amphotericin B^60^. Each gelatin filter was transferred to a 1.5 mL tube and dissolved with either ITM or VTM. After the dissolution of the gelatin filter, 140 or 400 μL aliquots were used for RNA extraction and RT-PCR. Collections were made at different distances and heights in relation to the patient’s position or convenience. Air sampling times were variable and are available in the Supplementary Material.

### Deposition samples

Deposition samples were collected as an expression of the natural deposition of particles due to gravity, according to physical factors (humidity, temperature). Deposition samples from individual households were collected using 50 mm Petri dishes with 2 mL of ITM. For the nursing home, 5 mL of VTM was used for each sample. Sampler’s location was dependent on local conditions. Petri dishes were placed on the top of furniture, higher than the patient’s height, to avoid contamination with natural droplet emissions.

### RNA extraction and RT-PCR

After collection, all samples were sent within 4 h to the laboratory, where virus inactivation and RNA extraction were performed immediately, followed by SARS-CoV-2 detection using reverse transcription polymerase chain reaction (RT-PCR). Virus inactivation and RNA extraction were performed using the GeneJet RNA viral extraction kit (Thermo Fisher Scientific, Waltham, MA, USA). SARS-CoV-2 RNA was detected using the SARS-CoV-2 RT-PCR protocol of the Institut Charité (Universitätsmedizin Berlin, Institute of Virology, Germany), as previously described65. The RT-PCR reaction was carried out in a LightCycler 2.0 (Roche, Basel, Switzerland), using a SensiFast Probe No-ROX one-step reverse transcription kit (Bioline, Meridian Biosciences, Cincinnati, OH, USA), according to the manufacturer’s protocol. Each 20-μL reaction mixture contained 5 μL of RNA, 10 μL of 2X SensiFAST Probe One-Step Mix, primer and probes, 0.2 μL of reverse transcriptase, and 0.4 μL of RiboSafe RNase Inhibitor. The thermal cycling conditions in RT-PCR assays were as follows: reverse transcription at 45 °C for 10 min, denaturation at 95 °C for 2 min, 45 cycles of amplification with denaturation at 95 °C for 5 s, annealing at 60 °C for 20 s, and extension at 72 °C for 15 s.

All laboratory procedures and sampling were conducted according to good practices and in compliance with Portuguese legislation for SARS-CoV-2 sampling and sample transportation as well as laboratory good practices according to CDC practices and as BSL-2 certified facility for SARS-CoV-2 detection by the Portuguese Authorities. Negative controls were obtained from non-exposed VTM or ITM. In the present study, all negative controls showed no amplification of unspecific products.

### SARS-CoV-2 viability assays

Air samples positive for viral RNA and collected using VTM were used to study SARS-CoV-2 viability. To validate if captured viral particles were viable, the presence of infectious particles was assessed qualitatively by monitoring host cells for occurring cytopathic effect (CPE)^62,63^. Then, SARS-CoV-2 identity was verified by RT-PCR of cell supernatants. Briefly, gelatin filters were dissolved in VTM and used for infection of Vero cells (ATCC, CCL-81), which were seeded one day prior to inoculation in T25 flasks. Cells were seeded in a growth medium (DMEM supplemented with 10% HI-FBS, 50 U/mL penicillin, 50 μg/mL streptomycin, and 2 mM glutamine), at a number to reach ~ 80–90% confluency the following day. The next day, cells were infected with 1 mL of sample inoculum and incubated for 90 min with gentle mixing every 15 min at 37 °C with 5% CO_2_. Subsequently, supernatants were removed and substituted by 8 mL of fresh maintenance medium (DMEM supplemented with 2.5% HI-FBS, 50 U/mL penicillin, 50 μg/mL streptomycin, and 2 mM glutamine). After 72 h of incubation at 37 °C with 5% CO_2_, supernatants were collected from each flask and frozen at − 80 °C in 1 mL aliquots. Cells were checked for the occurrence of CPE under an inverted microscope, and then fixed with 4% formaldehyde in phosphate buffered saline and stained with 0.1% toluidine blue. When there was no visible CPE after 72 h, 1 mL of supernatant was thawed and used for inoculation of fresh Vero cells seeded in T25 flasks, repeating the process up to 3 times (3 passages). Supernatants from cultures with visible CPE were thawed and treated with NVL lysis buffer (Nzytech, Lisbon, Portugal) to extract viral RNA, according to the manufacturer’s protocol. Then, they were analyzed through RT-PCR, as previously described. All experiments involving culturing of SARS-CoV-2 were performed in a certified Biosafety Level 3 (BSL-3) laboratory, following international and internal safety guidelines.

### Ethical approval

The project was approved by the Ethics Committee of the Faculty of Medicine of the University of Coimbra (CE-117/2020), performed under good clinical practice standards, and complying with the ethical precepts in the Helsinki Declaration and updates, including the Oviedo Agreement. The aims of the project and the commitments required were explained using an information brochure and all participants signed a statement of informed consent. All data were anonymized in the database, in accordance with the General Data Protection Regulation (UE) 2016/679. Patients with cancer, incidence of any immunological disease, and/or critical condition were excluded from this study.

## Supplementary Information

Supplementary Information.

## Data Availability

All data generated or analyzed during this study are included in this published article and its Supplementary Information files (Supplementary Tables S1, S2).
